# Laparoscopic hepatectomy for hepatocellular carcinoma in patients with clinically significant portal hypertension: a systematic review and meta-analysis

**DOI:** 10.1186/s12957-023-03264-7

**Published:** 2024-01-03

**Authors:** Zhi-qiang Xiang, Ya-chen Wu, Xi-lin Qu, Dan-jie Luo, Hao Liang, Sajid Ameer, Zhang-tao Long, Xiao-ming Dai, Zhu Zhu

**Affiliations:** 1 Department of Hepatobiliary Surgery, The First Affiliated Hospital, Hengyang Medical School, University of South China, Hengyang, 421001 Hunan China; 2 Hengyang Medical School, University of South China, Hengyang, 421001 Hunan China; 3 Department of Education and Training, The First Affiliated Hospital, Hengyang Medical School, University of South China, Hengyang, 421001 Hunan China

**Keywords:** Hepatocellular carcinoma, Cirrhosis, Portal hypertension, Laparoscopic hepatectomy, Meta-analysis

## Abstract

**Objective:**

To compare the effects of laparoscopic hepatectomy (LH) on the short-term and long-term outcomes in hepatocellular carcinoma (HCC) patients with and without clinically significant portal hypertension (CSPH).

**Methods:**

A systematic literature search of the PubMed, EMBASE, and Cochrane databases was performed for articles published from inception to March 1, 2023. Meta-analysis of surgical and oncological outcomes was performed using a random effects model. Data were summarized as mean difference and risk ratio with 95% confidence intervals.

**Results:**

Five cohort studies with a total of 310 HCC patients were included (CSPH 143; Non-CSPH 167). In terms of surgical outcomes, estimated blood loss and the length of hospital stay were significantly lower in the Non-CSPH group than in the CSPH group. There were no significant differences between the two groups regarding other surgical outcomes, including the operative time, ratio of conversion to open surgery, and overall complication rate. In addition, there were also no significant differences between the two groups regarding the oncological outcomes, such as 1-, 3-, and 5-year overall survival.

**Conclusions:**

HCC patients with and without CSPH who underwent LH had comparable surgical and oncological outcomes. LH is a safe and effective treatment for HCC patients with CSPH under the premise of rational screening of patients.

**Supplementary Information:**

The online version contains supplementary material available at 10.1186/s12957-023-03264-7.

## Introduction

Hepatocellular carcinoma (HCC) is the fifth most common and the third most deadly malignancy worldwide [1]. An estimated 905,700 cases were diagnosed with primary liver cancer and 830,200 patients died from the disease worldwide in 2020 [2]. Cirrhosis is currently considered the main precancerous lesion of primary HCC, and cirrhotic patients with clinically significant portal hypertension (CSPH) are more likely to develop HCC [3]. Due to the dearth of efficient radical treatments, the long-term prognosis of HCC patients with CSPH is poor, with a 5-year overall survival (OS) being less than 50% [4]. Up to now, there has been no uniform guideline for the treatment of HCC with CSPH due to the complex conditions of the patients and the great difficulty of the surgery.

According to the Barcelona Clinic Liver Cancer (BCLC) staging classification, guidelines by the European Association for the Study of the Liver and the American Association for the Study of Liver Diseases concluded that surgical resection is not recommended for HCC patients with CSPH [5, 6]. In recent years, increasing reports have demonstrated hepatectomy as a feasible and effective treatment for HCC patients with CSPH [7, 8]. With the rapid development of laparoscopic techniques, laparoscopic hepatectomy (LH) has been widely applied in HCC patients. It exhibits a superior short-term prognosis, including less blood loss, lower complication rate, and shorter hospital stay, while a similar long-term prognosis, compared with traditional open hepatectomy (OH) [9, 10]. However, there is disagreement regarding the surgical and oncological outcomes of LH in HCC patients with CSPH [11–15], and its safety and efficacy also remain controversial.

To address these above issues, we conducted a meta-analysis to analyze the surgical and oncological outcomes of LH and systematically evaluate the safety and efficacy of LH in HCC patients with CSPH. Our study might provide high-level evidence for those patients during surgical decision-making.

## Data and methods

The study followed the Preferred Reporting Items for Systematic Reviews and Meta-Analysis Statement (PRISMA) 2020 statement [16] and Assessing the Methodological Quality of Systematic Reviews (AMSTAR) guideline [17] (see Supplementary material for details). The review protocols were registered on PROSPERO (International Prospective Register of Systematic Reviews, number, CRD4202338799, https://www.crd.york.ac.uk/PROSPERO/display_record.php?RecordID=387,992).

### Search strategy

The PubMed, EMBASE, and Cochrane library databases were systematically searched for articles published from inception to March 1, 2023, using Medical Subject Headings (MeSH) combined with other keywords. Search terms used were as follows: hepatocellular carcinoma; portal hypertension; laparoscopy; hepatectomy; liver resection; laparoscopic hepatectomy and laparoscopic liver resection. The search strategy was established to answer the following research question: In patients with HCC (population), compared with non-CSPH (intervention and comparison), how CSPH can affect the surgical and oncological outcome(s)?

### Inclusion and exclusion criteria

#### Inclusion criteria (PICOS)

*Population:* patients diagnosed with primary HCC and underwent laparoscopic hepatectomy for curative intent.

*Interventions:* with CSPH.

*Comparisons:* without CSPH.

*Outcomes:* surgical outcomes such as estimated blood loss, operative time, conversion rate to open surgery, overall complication rate, and length of hospital stay, as well as oncological outcomes including overall survival.

*Study design:* prospective or retrospective study.

### Exclusion criteria

Exclusion criteria were as follows: (1) studies not reported in English; (2) studies with incomplete and invalid outcome indicators; (3) case reports, reviews, guidelines, conference abstracts, and expert consensus; (4) duplicate publication.

### Study selection and data extraction

Two investigators (X.Z.Q and W.Y.C) performed literature screening, data extraction, and cross-validation independently. Titles and abstracts were read first at the time of literature screening to remove obviously irrelevant studies. The full texts were further read to determine whether the article should be included or not. Any disagreements between the two reviewers were resolved through discussion with a third researcher (Z.Z).

The data extracted were (1) general characteristics: name of the first author, publication year, study period, nationality, study design, diagnostic criteria for CSPH, and number of cases in each study; (2) outcomes of interest: surgical outcomes including estimated blood loss, operative time, conversion rate to open surgery, overall complication rate, and length of hospital stay, as well as oncological outcomes such as 1-, 3-, and 5-year overall survival (OS). Survival data were extracted from Kaplan–Meier curves with Engauge Digitizer 11.3 (http://markummitchell.github.io/engauge-digitizer).

### Quality assessment

The two reviewers independently assessed the risk of bias in the included articles and cross-validated the results. The study quality was evaluated using the Newcastle Ottawa Scale (NOS) [18]. The score ranged from 0 to 9 points. Literature was defined as high-quality with a total score ≥ 7, and literature with a total score ≥ 6 was qualified to be included.

### Statistical analysis

Statistical analysis was performed using the RevMan 5.3 software. Continuous variables were assessed by mean difference (MD) with 95% confidence intervals (CI). If necessary, the estimation approach developed by Hozo et al. [19] was used to determine the mean and standard deviation. Dichotomous and survival-related variables were evaluated with the risk ratio (RR) with 95% CI [20]. The random effects model was adopted for the meta-analysis. Heterogeneity between studies was estimated by Cochran *Q* test and Higgins *I*^2^ statistics, with 25% as low, 50% as moderate, and 75% as high heterogeneity. If a significant heterogeneity across studies was observed (*I*^2^ > 50%), the source of heterogeneity would be further analyzed, and the robustness of the final results was assessed using the sensitivity analysis (leave-one-out method). Publication bias was illustrated using funnel plots. A two-tailed *P* < 0.05 was considered statistically significant.

## Results

**Fig. 1:**
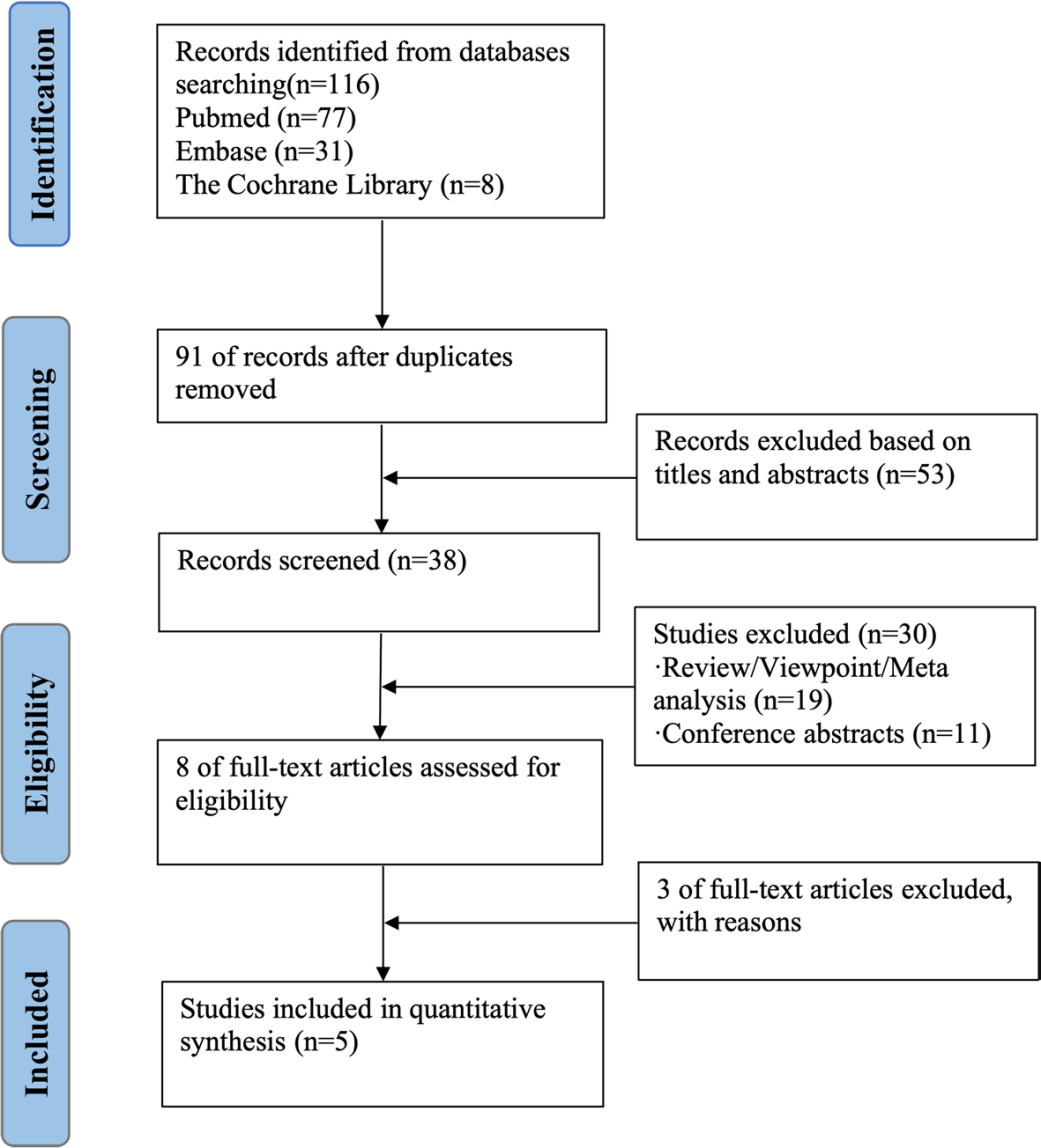
Flow chart of the selection process of studies used in this meta-analysis

Initially, 116 studies were identified, of which 25 were duplicate studies. Following a review of the titles and abstracts of the remaining 91 publications, 83 were excluded, of which 53 studies were irrelevant and 30 studies met the exclusion criteria. Three of the eight remaining studies were excluded after reading the full texts. Finally, five studies were included in the quantitative synthesis and meta-analysis [11–15]. Figure 1 shows the details of the literature search and selection process.

### Study characteristics

No RCTs were available for analysis, but 5 nonrandomized comparative studies were eligible for analysis, including one prospective study [13] and four retrospective studies [11, 12, 14, 15]. Two of these studies were from Spain, two from China, and one from France. A total of 310 HCC patients were included, of whom 143 (46.13%) had CSPH and 167 (53.87%) did not. The above studies are of high quality, according to the NOS, with scores ranging from 7 to 8 points. The general characteristics of the included studies are summarized in Table 1.

**Table 1:** Details of studies included in the meta-analysis

First author and years	Study period	Country	Type of study	Total sample	CSPH	Non-CSPH	NOS score
Guo 2022 [12]	2013–2018	China	Retrospective PSM	110	55	55	8
Lim 2019 [13]	2014–2017	France	Prospective PSM	45	18	27	7
Molina 2018 [14]	2005–2016	Spain	Retrospective	45	15	30	7
Robert 2020 [11]	2011–2018	Spain	Retrospective	62	31	31	8
Zheng 2021 [15]	2016–2019	China	Retrospective PSM	48	24	24	8

*PSM* propensity score matching, *BMI* body mass index, *ASA* American Society of Anesthesiologists, *AFP* alpha-fetoprotein, *ALT* alanine aminotransferase, *HCC* hepatocellular carcinoma, *HVPG* hepatic venous pressure gradient

### Surgical outcomes

**Fig. 2:**
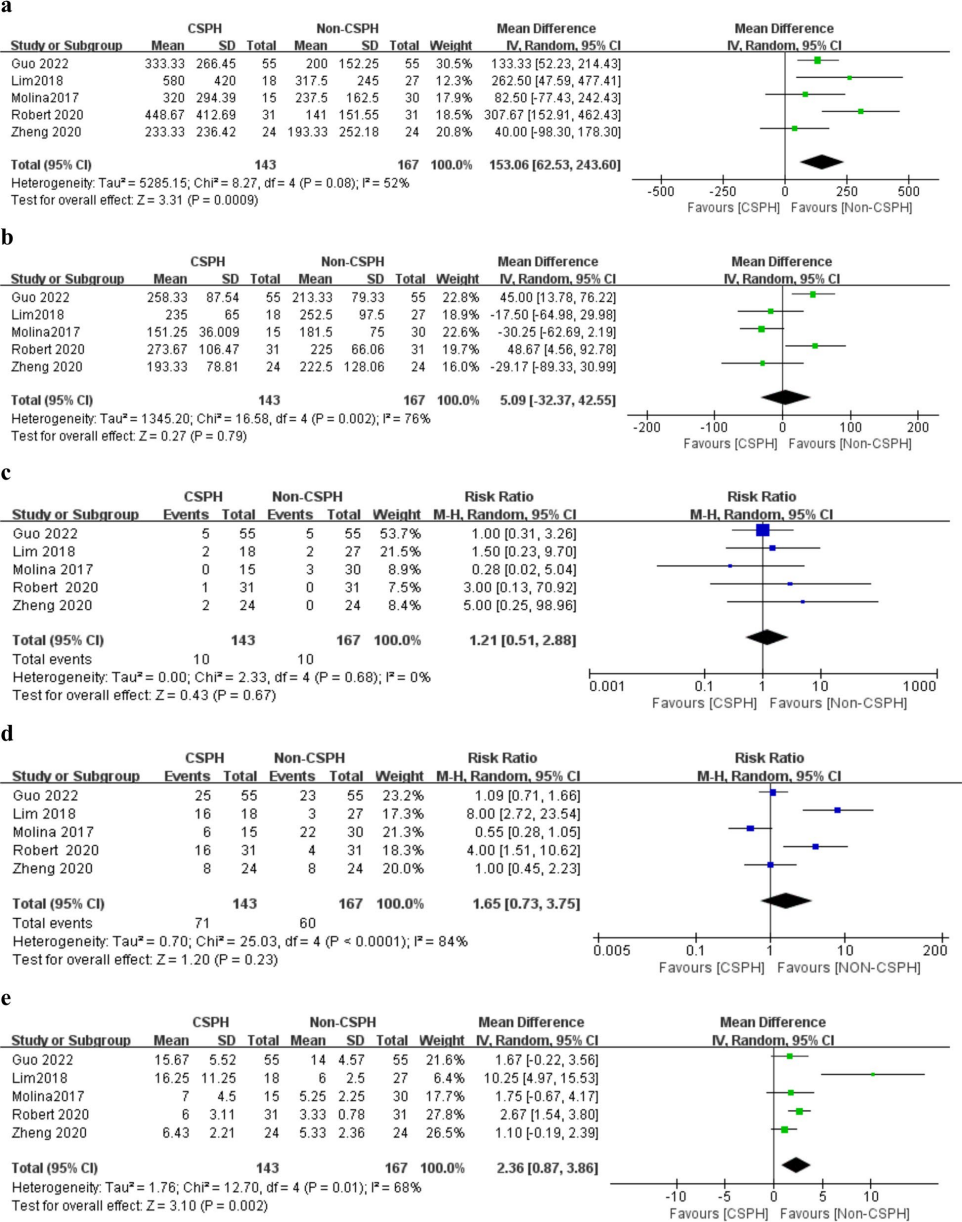
Forest plots of surgical outcomes, including **a** estimated blood loss, **b** operation time, **c** conversion to open surgery, **d** overall complications, and **e** length of hospital stay

The surgical outcomes were reported by all the five studies [11–15]. The estimated blood loss was significantly higher in the CPSH group than in the non-CSPH group (MD = 153.06, 95% CI = 62.53–243.60, *P* = 0.0009) (Fig. 2a). However, the results showed no statistically significant differences in the operative time, conversion rate to open surgery and overall complication rate between the two groups (MD = 5.09, 95% CI = -32.37–42.55, *P* = 0.79; RR = 1.21, 95% CI = 0.51-2.88, *P* = 0.67; RR = 1.65, 95% CI = 0.73–3.75, *P* = 0.23) (Fig. 2b–d). Moreover, the length of postoperative hospital stay was longer in the CPSH group than in the non-CSPH group (MD = 2.36, 95% CI = 0.87–3.86, *P* = 0.002) (Fig. 2e).

### Oncological outcomes

**Fig. 3:**
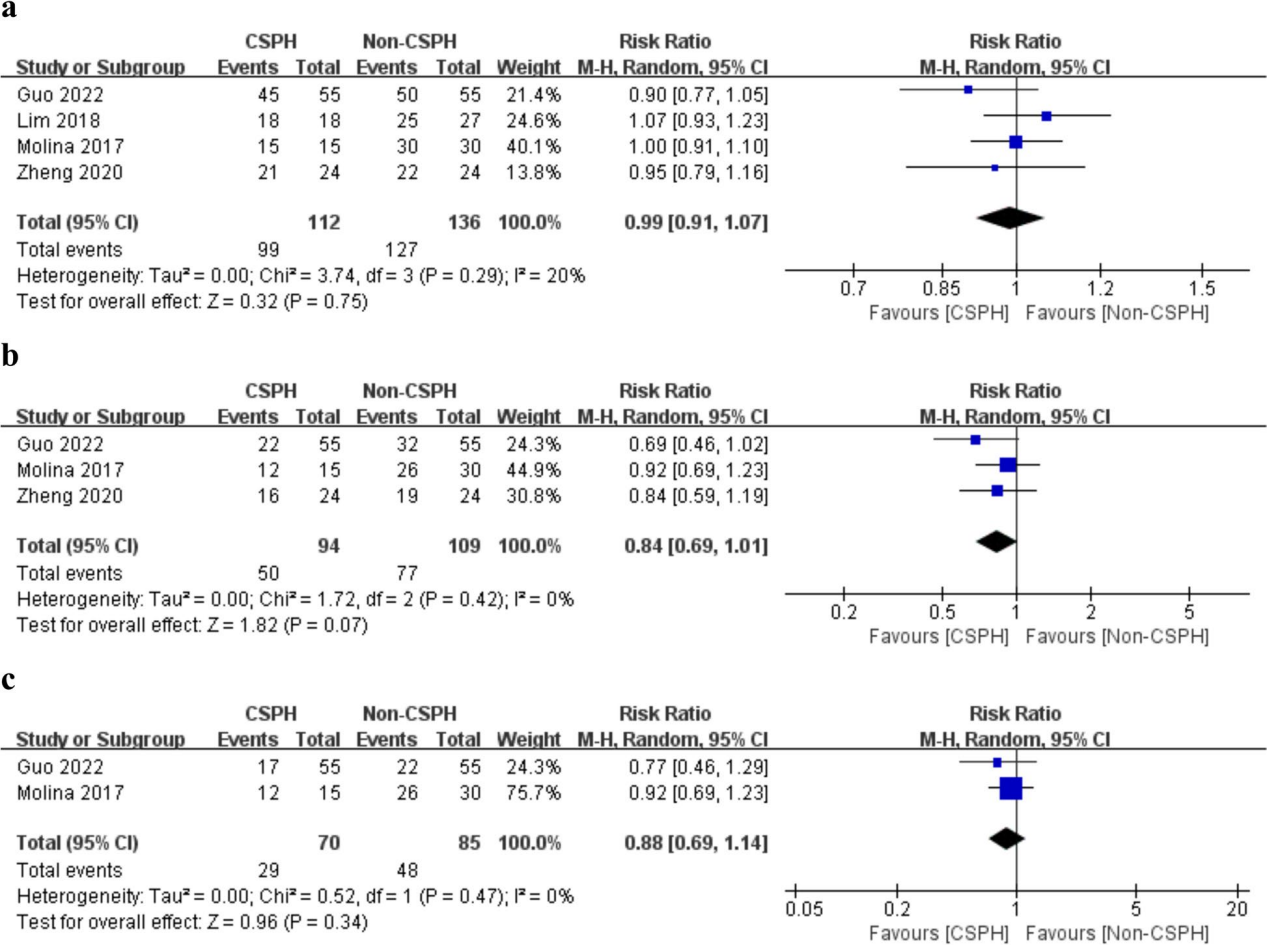
Forest plots of oncological outcomes, including **a** 1-year overall survival, **b** 3-year overall survival, and **c** 5-year overall survival

The 1-year OS was reported by four studies [12–15]. Besides, three studies reported 3-year OS [12, 14, 15], and two reported 5-year OS [12, 14]. The heterogeneity test showed no significant heterogeneity among the studies (*P* = 0.29, *I*^2^ = 20%; *P* = 0.42, *I*^2^ = 0%; *P* = 0.47, *I*^2^ = 0%). The results revealed no significant difference in 1-, 3-, and 5-year OS between the two groups (RR = 0.99, 95% CI = 0.91-1.07, *P* = 0.75; RR = 0.84, 95% CI = 0.69-1.01, *P* = 0.07; RR = 0.88, 95% CI = 0.69-1.14, *P* = 0.34, respectively) (Fig. 3a–c).

### Sensitivity analysis and publication bias

**Fig. 4:**
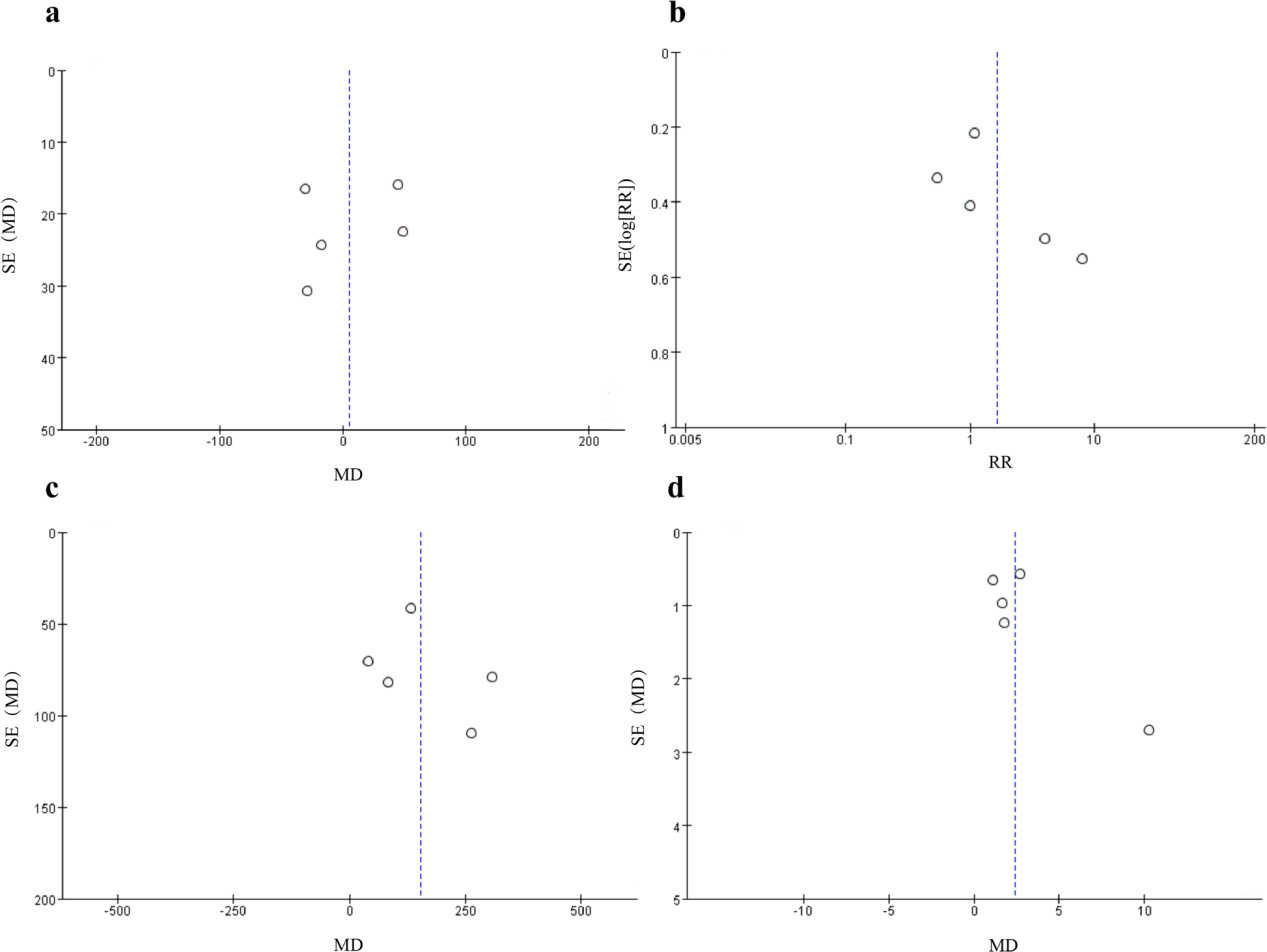
Funnel plots on surgical and oncological outcomes, including **a** operation time, **b** overall complication rate, **c** estimated blood loss, and **d** length of hospital stay

The Cochran *Q* test revealed high heterogeneity in operative time and overall complication rate (*P* = 0.002, *I*^2^ = 76%; *P* < 0.0001, *I*^2^ = 84%), moderate heterogeneity in estimated blood loss and length of hospital stay (*P* = 0.08, *I*^2^ = 52%; *P* = 0.01, *I*^2^ = 68%). The sensitivity analysis of the above results with significant heterogeneity was performed using the leave-one-out method, and the risk assessment level and significance level of the outcome indices remained unchanged. In terms of publication bias, we found no obvious publication bias, as illustrated by the constructed funnel plots (Fig. 4a–d).

## Discussions

The major finding of our study was that there was no significant difference in HCC patients undergoing LH with and without CSPH regarding surgical outcomes such as operative time, conversion rate to open surgery, and overall complication rate, as well as oncological outcomes including 1-, 3-, and 5-year OS. Nevertheless, estimated blood loss and length of hospital stay favored patients without CSPH. To our knowledge, this is the first systematic review and meta-analysis to evaluate the safety and efficacy of LH in HCC patients with CSPH. Previous meta-analyses concluded that HCC patients with CSPH have significantly higher postoperative complication rates and mortality and significantly lower OS than those without CSPH [21, 22]. These findings were inconsistent with our study, possibly due to the fact that in our study more patients received OH rather than LH.

In terms of surgical outcomes, patients in the CPSH group showed greater estimated blood loss and longer hospital stays than the Non-CSPH group for the following reasons. First, the blood loss would increase during hepatic parenchymal transection as a result of abundant peripheral collateral circulation and higher portal pressure in CSPH patients. Second, it was more challenging to control intraoperative bleeding in patients complicated with splenic thrombocytopenia or hepatic coagulation dysfunction [23]. Finally, HCC patients with CSPH generally had impaired liver function, which caused prolonged postoperative recovery frequently [24].

However, no significant difference between the two groups was found in operative time, conversion rate to open surgery, and overall complication rate. Independent risk factors for the conversion rate to open surgery during LH included large tumor size, extensive hepatectomy, cirrhosis, and portal hypertension [25–27]. Through a meta-analysis, Wang et al. revealed no significant difference in the conversion rate to open surgery between patients with and without cirrhosis performing LH [28]. This study was consistent with our findings and further supported our results. Additionally, studies have shown that operative time is an independent risk factor for complication rate in patients performing LH, with a 60% increase in the postoperative complication rate for each additional hour of operation time [29, 30]. Furthermore, several investigations have revealed that the postoperative complication rate following LH was higher in patients who were converted to OH than those who did not [31]. Therefore, our findings (i.e., comparable overall complication rate) might be attributed to comparable operative time and conversion to open surgery between the two groups.

Concerning the oncological outcomes, our study found equivalence in 1-, 3-, and 5-year OS between the CPSH and Non-CSPH groups. A recent meta-analysis suggested that CSPH is an independent risk factor for long-term OS in HCC patients after partial hepatectomy, with a negative impact on the 5-year OS. However, CSPH did not affect the long-term outcome in a subgroup of European HCC patients [32]. The reason might be that HCC patients in Europe are diagnosed at an early stage, and the selection criteria for surgery are stricter than those in Asia. Our meta-analysis found that the two groups had comparable oncological outcomes for the following reasons: (a) the number of cases from Europe or Asia was comparable in the included studies. (b) all Asian and some European studies used the propensity matching score to balance the baseline level and eliminate the selection bias among groups.

Our study also had several limitations: (a) The screening criteria of the patient were different, e.g., the diagnostic criteria of CSPH varied among the included studies. (b) The enrolled studies were mainly retrospective studies, and high-quality studies such as RCTs are lacking. (c) Most included studies are absent of long-term DFS; thus, the impact of CSPH on long-term outcomes such as recurrence and metastasis could not be further evaluated. Therefore, it is crucial to conduct prospective, multicenter, and long-term follow-up RCTs with a large sample size to determine the impact of LH on the surgical and oncological outcomes of HCC patients with CPSH.

## Conclusions

In summary, HCC patients with CSPH have surgical and oncological outcomes comparable to those without CSPH. Therefore, LH is a safe and effective treatment for HCC patients with CSPH on the assumption of reasonable screening of patients. HCC combined with CSPH may serve as a surgical indication for LH.

### Supplementary Information

**Additional file 1.** Flow chart.  PRISMA 2020 Checklist.  AMSTAR 2.  Search strategy.

## Data Availability

All data generated or analyzed during this study are included in this published article and its supplementary information files.
