# Supplementary material for: Laparoscopic hepatectomy for hepatocellular carcinoma in patients with clinically significant portal hypertension: a systematic review and meta-analysis
Source: World J Surg Oncol. 2024 Jan 3;22:3. doi: 10.1186/s12957-023-03264-7 (PMC10763288; doi:10.1186/s12957-023-03264-7)
Supplement: Supplementary file 1 — Additional file 1. Flow chart. PRISMA 2020 Checklist. AMSTAR 2. Search strategy. [file 12957_2023_3264_MOESM1_ESM.zip › Flow Chat.docx]

3 of full-text articles excluded, with reasons

Studies excluded (n=30),

·Review/Viewpoint/Meta analysis (n=19)

·Conference abstracts (n=11)

Records excluded based on titles and abstracts (n=53)

8 of full-text articles assessed for eligibility

91 of records after duplicates removed

Records screened (n=38)

Studies included in quantitative synthesis (n=5)

Records identified from databases searching(n=116)
Pubmed (n=77)
Embase (n=31)
The Cochrane Library (n=8)

**Included**

**Eligibility**

**Screening**

**Identification**
