# Supplementary material for: Laparoscopic hepatectomy for hepatocellular carcinoma in patients with clinically significant portal hypertension: a systematic review and meta-analysis
Source: World J Surg Oncol. 2024 Jan 3;22:3. doi: 10.1186/s12957-023-03264-7 (PMC10763288; doi:10.1186/s12957-023-03264-7)
Supplement: Supplementary file 1 — Additional file 1. Flow chart. PRISMA 2020 Checklist. AMSTAR 2. Search strategy. [file 12957_2023_3264_MOESM1_ESM.zip › Search strategy.docx]

Search strategy

Pubmed

#1 "Carcinoma, Hepatocellular"[MeSH Terms]

#2 (((((((((((((((((((Carcinomas, Hepatocellular[Title/Abstract]) OR (Hepatocellular Carcinomas[Title/Abstract])) OR (Liver Cell Carcinoma, Adult[Title/Abstract])) OR (Liver Cancer, Adult[Title/Abstract])) OR (Adult Liver Cancer[Title/Abstract])) OR (Adult Liver Cancers[Title/Abstract])) OR (Cancer, Adult Liver[Title/Abstract])) OR (Cancers, Adult Liver[Title/Abstract])) OR (Liver Cancers, Adult[Title/Abstract])) OR (Liver Cell Carcinoma[Title/Abstract])) OR (Carcinoma, Liver Cell[Title/Abstract])) OR (Carcinomas, Liver Cell[Title/Abstract])) OR (Cell Carcinoma, Liver[Title/Abstract])) OR (Cell Carcinomas, Liver[Title/Abstract])) OR (Liver Cell Carcinomas[Title/Abstract])) OR (Hepatocellular Carcinoma[Title/Abstract])) OR (Hepatoma[Title/Abstract])) OR (Hepatomas[Title/Abstract])) OR (liver neoplasms[Title/Abstract]))

#3（#1 OR #2）

#4"hypertension, portal"[MeSH Terms]

#5 (((((Hypertensions, Portal[Title/Abstract]) OR (Portal Hypertension[Title/Abstract])) OR (Portal Hypertensions[Title/Abstract])) OR (Cruveilhier-Baumgarten Syndrome[Title/Abstract])) OR (Cruveilhier Baumgarten Syndrome[Title/Abstract])) OR (Syndrome, Cruveilhier-Baumgarten[Title/Abstract])

#6（#4 OR #5）

#7"Laparoscopy"[MeSH Terms]

#8 ((((((((((((((((((Laparoscopies[Title/Abstract]) OR (Celioscopy[Title/Abstract])) OR (Celioscopies[Title/Abstract])) OR (Peritoneoscopy[Title/Abstract])) OR (Peritoneoscopies[Title/Abstract])) OR (Surgical Procedures, Laparoscopic[Title/Abstract])) OR (Laparoscopic Surgical Procedure[Title/Abstract])) OR (Procedure, Laparoscopic Surgical[Title/Abstract])) OR (Procedures, Laparoscopic Surgical[Title/Abstract])) OR (Surgery, Laparoscopic[Title/Abstract])) OR (Laparoscopic Surgical Procedures[Title/Abstract])) OR (Laparoscopic Surgery[Title/Abstract])) OR (Laparoscopic Surgeries[Title/Abstract])) OR (Surgeries, Laparoscopic[Title/Abstract])) OR (Laparoscopic Assisted Surgery[Title/Abstract])) OR (Laparoscopic Assisted Surgeries[Title/Abstract])) OR (Surgeries, Laparoscopic Assisted[Title/Abstract])) OR (Surgery, Laparoscopic Assisted[Title/Abstract])) OR (Surgical Procedure, Laparoscopic[Title/Abstract])

#9（#7 OR #8）

#10 ("1976/01/01"[Date - Publication] : "2022/03/01"[Date – Publication])

#11(#3 AND #6 AND #9 AND #10)

Embase

#1 'liver cell carcinoma'/exp

#2 'carcinoma，hepatocellular':ab,ti OR 'carcinomas, hepatocellular':ab,ti OR 'hepatocellular carcinomas':ab,ti OR 'liver cell carcinoma, adult':ab,ti OR 'liver cancer, adult':ab,ti OR 'adult liver cancer':ab,ti OR 'adult liver cancers':ab,ti OR 'cancer, adult liver':ab,ti OR 'cancers,adult liver':ab,ti OR 'liver cancers,adult':ab,ti OR 'carcinoma, liver cell':ab,ti OR 'carcinomas, liver cell':ab,ti OR 'cell carcinoma,liver':ab,ti OR 'cell carcinomas, liver':ab,ti OR 'liver cell carcinomas':ab,ti OR 'hepatocellular carcinoma':ab,ti OR 'hepatoma':ab,ti OR 'hepatomas':ab,ti OR ' liver neoplasms':ab,ti

#3 (#1 OR #2)

#4'Hypertension, Portal':ab,ti or 'Hypertensions, Portal':ab,ti or 'Portal Hypertension':ab,ti or 'Portal Hypertensions':ab,ti or 'Cruveilhier-Baumgarten Syndrome':ab,ti or 'Cruveilhier Baumgarten Syndrome':ab,ti or 'Syndrome, Cruveilhier-Baumgarten':ab,ti

#5'laparoscopy':ab,ti OR 'laparoscopies':ab,ti OR 'celioscopy':ab,ti OR 'celioscopies':ab,ti OR 'peritoneoscopy':ab,ti OR 'peritoneoscopies':ab,ti OR 'surgical procedures, laparoscopic':ab,ti OR 'laparoscopic surgical procedure':ab,ti OR 'procedure, laparoscopic surgical':ab,ti OR 'procedures, laparoscopic surgical':ab,ti OR 'surgery, laparoscopic':ab,ti OR 'laparoscopic surgical procedures':ab,ti OR 'laparoscopic surgery':ab,ti OR 'laparoscopic surgeries':ab,ti OR 'surgeries, laparoscopic':ab,ti OR 'laparoscopic assisted surgery':ab,ti OR 'laparoscopic assisted surgeries':ab,ti OR 'surgeries, laparoscopic assisted':ab,ti OR 'surgery, laparoscopic assisted':ab,ti OR 'surgical procedure, laparoscopic':ab,ti

#6 (#4 AND #5 AND #3 AND [01-01-1976]/sd NOT [01-03-2022])

Cochrane Library

#1 MeSH descriptor: [Carcinoma, Hepatocellular] explode all trees

#2 (Carcinomas, Hepatocellular):ab,kw or (Hepatocellular Carcinomas):ab,kw or (Liver Cell Carcinoma, Adult):ab,kw or (Liver Cancer, Adult):ab,kw or (Adult Liver Cancer):ab,kw or (Adult Liver Cancers):ab,kw or (Cancer, Adult Liver):ab,kw or (Cancers,Adult Liver):ab,kw or (Liver Cell Carcinoma):ab,kw or (Liver Cancers,Adult):ab,kw or (Carcinoma, Liver Cell):ab,kw or (Carcinomas, Liver Cell):ab,kw or (Cell Carcinoma,Liver):ab,kw or (Cell Carcinomas, Liver):ab,kw or (Liver Cell Carcinomas):ab,kw or (Hepatocellular Carcinoma):ab,kw or (Hepatomas):ab,kw or (Hepatoma):ab,kw or (liver neoplasms):ab,kw

#3 (#1 OR #2)

#4(Hypertension, Portal):ti,ab,kw OR (Hypertensions, Portal):ti,ab,kw OR (Portal Hypertension):ti,ab,kw OR (Portal Hypertensions):ti,ab,kw OR (Cruveilhier-Baumgarten Syndrome):ti,ab,kw OR (Cruveilhier Baumgarten Syndrome):ti,ab,kw OR (Syndrome, Cruveilhier-Baumgarten):ti,ab,kw

#5(Laparoscopy ):ti,ab,kw OR (Laparoscopies):ti,ab,kw OR (Celioscopy):ti,ab,kw OR (Celioscopies):ti,ab,kw OR (Peritoneoscopy):ti,ab,kw OR (Peritoneoscopies):ti,ab,kw OR (Surgical Procedures, Laparoscopic):ti,ab,kw OR (Laparoscopic Surgical Procedure):ti,ab,kw OR (Procedure, Laparoscopic Surgical):ti,ab,kw OR (Procedures, Laparoscopic Surgical):ti,ab,kw OR (Surgery, Laparoscopic):ti,ab,kw OR (Laparoscopic Surgical Procedures):ti,ab,kw OR (Laparoscopic Surgery):ti,ab,kw OR (Laparoscopic Surgeries):ti,ab,kw OR (Surgeries, Laparoscopic):ti,ab,kw OR (Laparoscopic Assisted Surgery):ti,ab,kw OR (Laparoscopic Assisted Surgeries):ti,ab,kw OR (Surgeries, Laparoscopic Assisted):ti,ab,kw OR (Surgery, Laparoscopic Assisted):ti,ab,kw OR (Surgical Procedure, Laparoscopic):ti,ab,kw

#6 (#4 AND #5 AND #3)

With Cochrane Library publication date from Jan 1976 to Mar 2023,in Trials
